# Mapping the Atomistic Structure of Graded Core/Shell Colloidal Nanocrystals

**DOI:** 10.1038/s41598-017-11996-2

**Published:** 2017-09-15

**Authors:** Maksym Yarema, Yunhua Xing, Rainer T. Lechner, Lukas Ludescher, Nikola Dordevic, Weyde M. M. Lin, Olesya Yarema, Vanessa Wood

**Affiliations:** 1 Laboratory for Nanoelectronics, Department of Information Technology and Electrical Engineering, ETH Zurich, CH-8092 Zurich, Switzerland; 2 Institute of Physics, Montanuniversitaet Leoben, A-8700 Leoben, Austria

## Abstract

Engineering the compositional gradient for core/shell semiconductor nanocrystals improves their optical properties. To date, however, the structure of graded core/shell nanocrystal emitters has only been qualitatively described. In this paper, we demonstrate an approach to quantify nanocrystal structure, selecting graded Ag-In-Se/ZnSe core/shell nanocrystals as a proof-of-concept material. A combination of multi-energy small-angle X-ray scattering and electron microscopy techniques enables us to establish the radial distribution of ZnSe with sub-nanometer resolution. Using *ab initio* shape-retrieval analysis of X-ray scattering spectra, we further determine the average shape of nanocrystals. These results allow us to generate three-dimensional, atomistic reconstructions of graded core/shell nanocrystals. We use these reconstructions to calculate solid-state Zn diffusion in the Ag-In-Se nanocrystals and the lattice mismatch between nanocrystal monolayers. Finally, we apply these findings to propose design rules for optimal shell structure and record-luminescent core/shell nanocrystals.

## Introduction

Due to efficient and tunable emission properties, semiconductor core/shell nanocrystals (NCs) are of high technological importance for solid-state lighting and bio-medical applications^1,2^. Optical properties of core/shell nanomaterials can be improved by engineering a graded protective shell^3,4^. Relaxed lattice interfaces between core and shell materials lead to smaller density of interfacial defects and lower Auger non-radiative recombination^5,6^. At the same time, the absence of an atomically defined interface makes determination of the atomic structure of graded core/shell NCs a complicated task.

Graded shells have been realized through mixing of cations (*e.g*., ZnSe/CdSe NCs)^7^, anions (*e.g*., CdSe/CdS NCs)^6^, or for both atomic sites (*e.g*., CdSe/ZnS NCs)^8^. Research efforts on graded core/shell NCs have focused primarily on obtaining high luminescent efficiencies but not on the composition and structural characteristics of the graded shell. Typically, the alloying of two materials into graded core/shell NCs is indirectly proven by optical properties, such as blue shift of emission wavelength or change of luminescent lifetime and luminescence efficiency^9,10^. Insight into the composition and structure of a graded design can be obtained by carefully designed experiments. For example, successive ionic layer adsorption and reaction (SILAR) synthesis allows one to estimate the atomic gradients^11^. Alternatively, electron microscopy results of reaction aliquots can provide measures of size and composition during growth of core/shell NCs, for subsequent reconstruction of the composition gradient. However, these strategies cannot be applied for numerous scenarios, which include fast formation of graded shell or post-synthetic annealing of core/shell NCs^3,12^.

**Figure 1:**
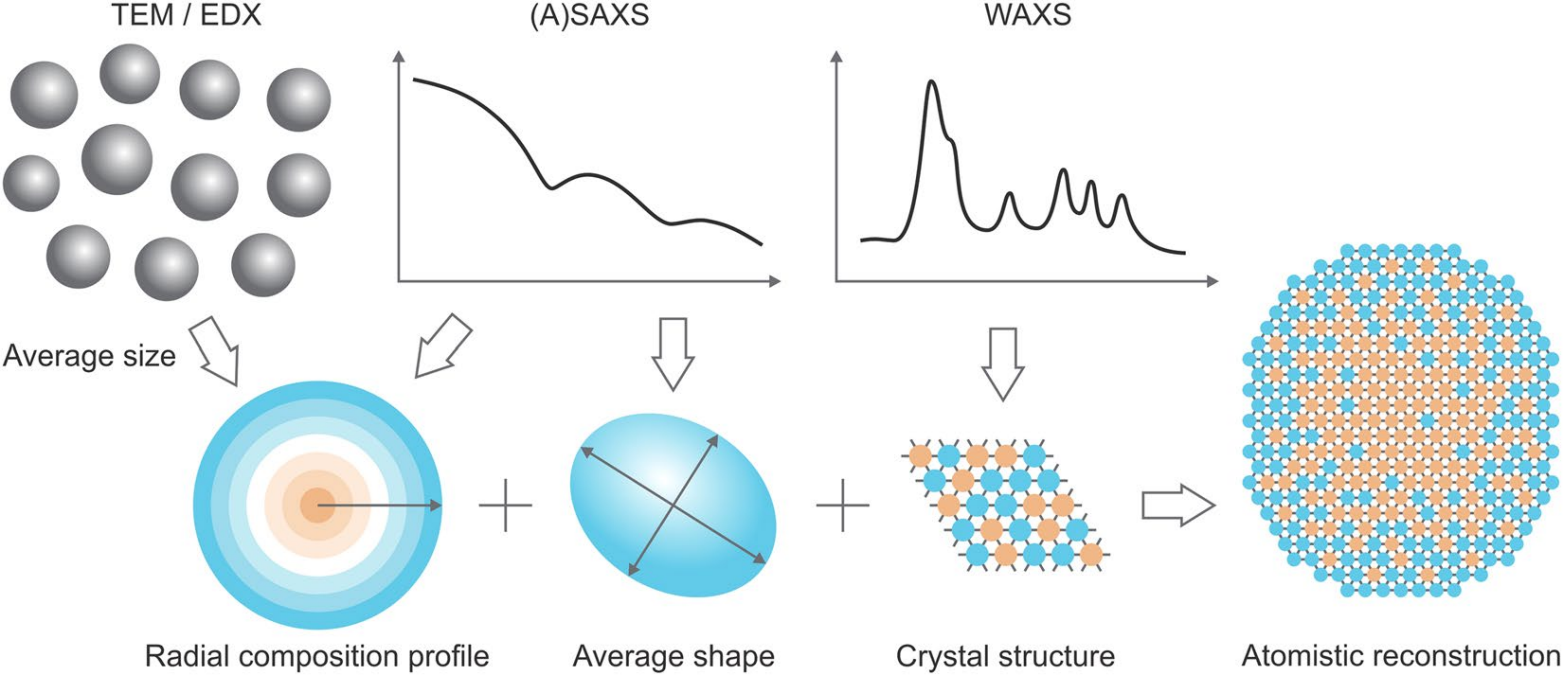
Approach for atomistic reconstructions. The structure of graded core/shell nanocrystals can be resolved on atomic level, using a combination of electron microscopy and X-ray scattering methods. TEM denotes transmission electron microscopy; EDX – energy-dispersive X-ray spectroscopy; (A)SAXS – (anomalous) small-angle X-ray scattering; WAXS – wide-angle X-ray scattering.

In this paper, we present a method to obtain atomic reconstructions of graded core/shell NCs using multi-energy X-ray scattering techniques^13,14^. Our approach is illustrated in Fig. 1. We determine the average radial distribution of elements with sub-nanometer resolution using anomalous small-angle X-ray scattering (ASAXS) analysis, supported by electron microscopy. This information is combined with retrievals of the average shape of NCs obtained from *ab initio* shape retrieval from SAXS data^15^ and crystal structure information from wide-angle X-ray scattering (WAXS) to create atomic reconstructions of the NCs. We show that this detailed understanding of structure enables quantitative studies of solid-state diffusion in the NCs and lattice relaxation at the core/shell interface. Finally, we link these results to the luminescence efficiency of graded core/shell NCs.

**Figure 2:**
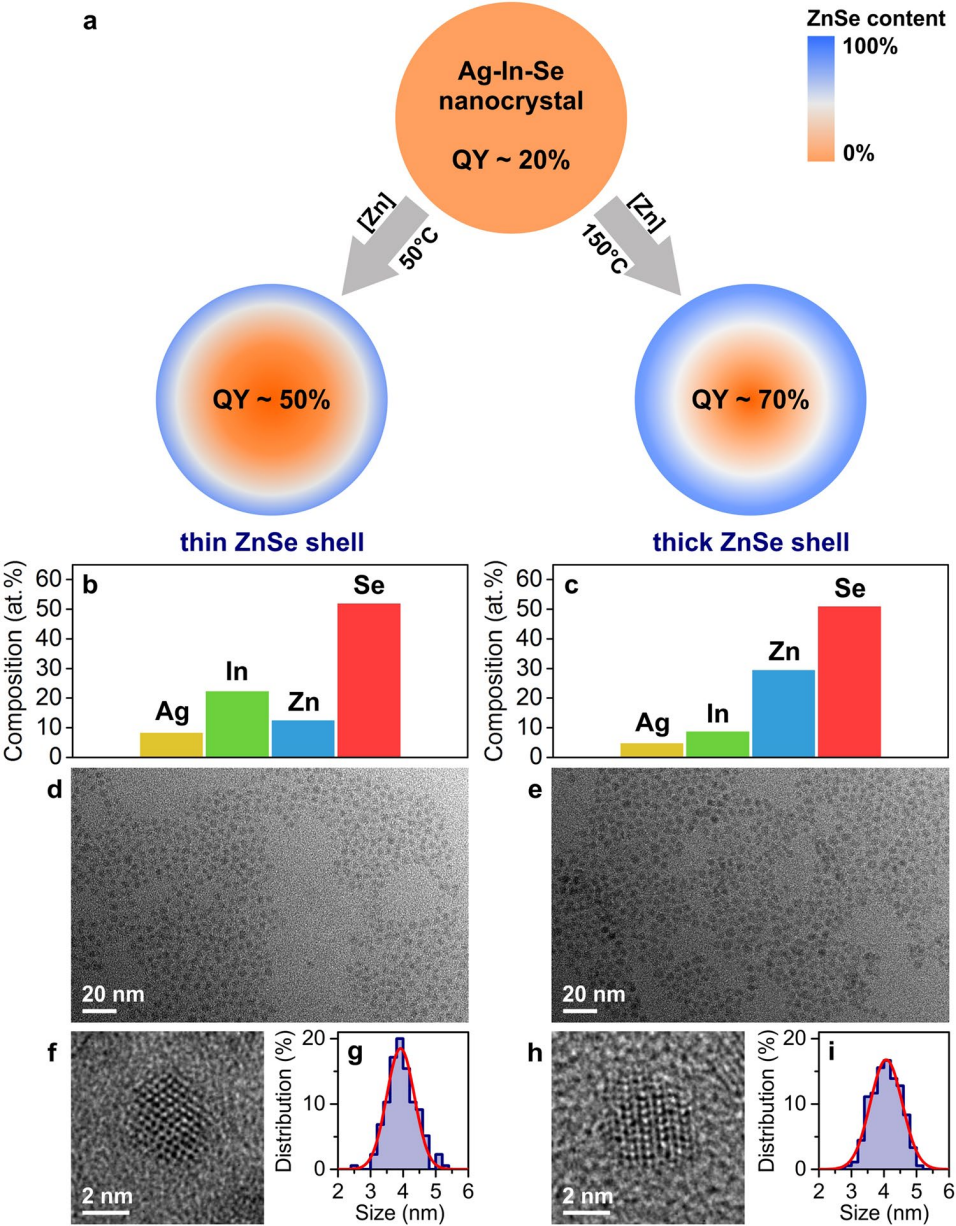
Model system: graded Ag-In-Se/ZnSe core/shell nanocrystals. (**a**) Schematic illustration of the ZnSe shell growth process. Two investigated samples with distinct ZnSe atomic profiles are prepared at different shell growth temperatures. QY denoted luminescence quantum yield. (**b**,**c**) Energy dispersive X-ray spectra quantifications, (**d**,**e**) representative TEM images, (**f**,**h**) high-resolution TEM images, and (**g**,**i**) size distributions of investigated Ag-In-Se/ZnSe core/shell nanocrystals. Data for thin ZnSe shell sample are shown in the left panel and for thick ZnSe shell sample in the right panel.

As a model system, we choose the case of Ag-In-Se/ZnSe core/shell NCs. Upon growth of a ZnSe shell, the luminescence efficiency increases, with current reports of up to 70% quantum yield (QY) for this system^16^. Owing to high miscibility between I-III-VI and II-VI semiconductors and to a large number of cationic defects, which facilitate diffusion^17,18^, this system yields graded core/shell structures. Starting from the same Ag-In-Se cores, we employ two different shell growth temperatures^7^. Due to the strong temperature dependence of cation-exchange processes, we obtain distinct radial profiles of ZnSe (Fig. 2), which we refer to as *thin ZnSe shell* NCs (*T*_*growth*_ = 50 °C) and *thick ZnSe shell* NCs (*T*_*growth*_ = 150 °C). In this paper, we quantify the radial distributions of Zn with a resolution of ~0.4 nm, determine that the NCs exhibit on average an ellipsoidal shape, and associate the elongated axis with the crystal structure of graded Ag-In-Se/ZnSe NCs. Better understanding of the structure of graded core/shell NCs helps to explain the relative luminescence efficiencies. The Ag-In-Se/ZnSe NCs, prepared at a higher temperature of 150 °C (Fig. 2a), exhibit a high degree of alloying due to efficient Zn diffusion into the NCs during growth. This sample is fully covered with a protective ZnSe shell, showing small lattice mismatch at the core/shell interface. In contrast, low temperature growth of the ZnSe shell (*T*_*growth*_ = 50 °C) leads to inefficient Zn diffusion, incomplete surface protection, and strained core/shell structure. In accordance with this structural analysis, the luminescence efficiency of *thick ZnSe shell* NCs is higher than that of *thin ZnSe shell* NCs.

## Results

### Obtaining a radial composition profile

Anomalous small-angle X-ray scattering (or ASAXS) is sensitive to element-specific variations in a material^13^. This is achieved by measuring at energies around the absorption edge of the element of interest. In the case of graded Ag-In-Se/ZnSe core/shell NCs, Zn is chosen as the anomalous scatterer. As the incident energy of X-rays approaches the K-absorption edge of Zn, core electrons on the Zn atoms become resonant with the incident photons. Consequently, fewer electrons contribute to the scattering, which results in a lower intensity and a shift of the scattering curves. ASAXS spectra reflect both the energy-independent (*i.e*., elastic scattering, typical in SAXS) as well as the anomalous scattering (Figure S1). Therefore, the scattering intensity is a function of the total and atomic-specific (*i.e*., Zn) electron density contrasts between the scatterer (*i.e*., the nanocrystal) and the medium (*i.e*., solvent, polymer matrix *etc*.)^13^. Other fitting parameters of typical SAXS model include shape, size, and size distribution function of the scattering particles.

For core/shell NCs with an abrupt core/shell interface, additional parameters are needed to fit ASAXS spectra: the electronic density contrast between core and shell materials (both total and element-specific) as well as geometrical variables (shell thickness, shape of the core region, *etc*.). Indeed, this has been demonstrated for PbS/CdS core/shell NCs, systematically varying core size and shell thickness^14^.

Core/shell NCs with gradient-shell morphology represent an even more complex object for ASAXS analysis, requiring a large number of parameters in the fitting model (e.g., more than 20 fitting parameters for 5-shell ASAXS model)^19^. To reduce this complexity, we develop a simple yet representative ASAXS model for the structure of graded Ag-In-Se/ZnSe core/shell scatterer, which we refer to as the *equispaced multi-shell ASAXS model*. The model assumes that Zn diffusion during the cation-exchange process is isotropic^7,10,14^. The graded core/shell NC is decomposed into concentric regions, each having the same thickness (*i.e*., equispaced multi-shells). The ZnSe content is different in each shell can differ according to the radial composition profile of the graded core/shell NC. A full mathematical representation of the model is provided in the Supplementary Information.

To reduce the number of fitting parameters in the equispaced multi-shell ASAXS model, we leverage several electron microscopy techniques (Fig. 2b–i). Energy-dispersive X-ray (EDX) spectroscopy reveals the average atomic composition of the NCs. We find that the Ag:In atomic ratio remains around 2 for both thin-shell and thick-shell Ag-In-Se/ZnSe NCs (Fig. 2b,c), which means that the composition of NCs can be represented as (Ag_2/7_In_4/7_Se)_1−x_(ZnSe)_x_ with variable *x (i.e*., as a solid solution of Ag_2_In_4_Se_7_ and ZnSe). Based on these assumptions, the total and Zn-specific electron densities can be presented as a function of *x* in (Ag_2/7_In_4/7_Se)_1−x_(ZnSe)_x_ (more details in the Supplementary Information, Figures S2 and S3). Analysis of transmission electron microscopy (TEM) images provides independent information on NC size and shape. Here, we first make the approximation that the graded Ag-In-Se/ZnSe core/shell NCs are spherical^16^, and in the section below, show how the results change if the NCs are assumed to be ellipsoidal. From TEM, we define size distribution functions as Gaussians with expectations of 3.9 nm and 4.1 nm and standard deviations of 0.43 nm and 0.49 nm for thin-shell and thick-shell Ag-In-Se/ZnSe NCs, respectively (Fig. 2d–i). We use these data as fixed parameters in the equispaced multi-shell ASAXS model. Eventually, the ZnSe content, *x*, in (Ag_2/7_In_4/7_Se)_1−x_(ZnSe)_x_ composition becomes a single fitting parameter for each shell. Thus, the number of fitting parameters in the model is simply the number of shells.

**Figure 3:**
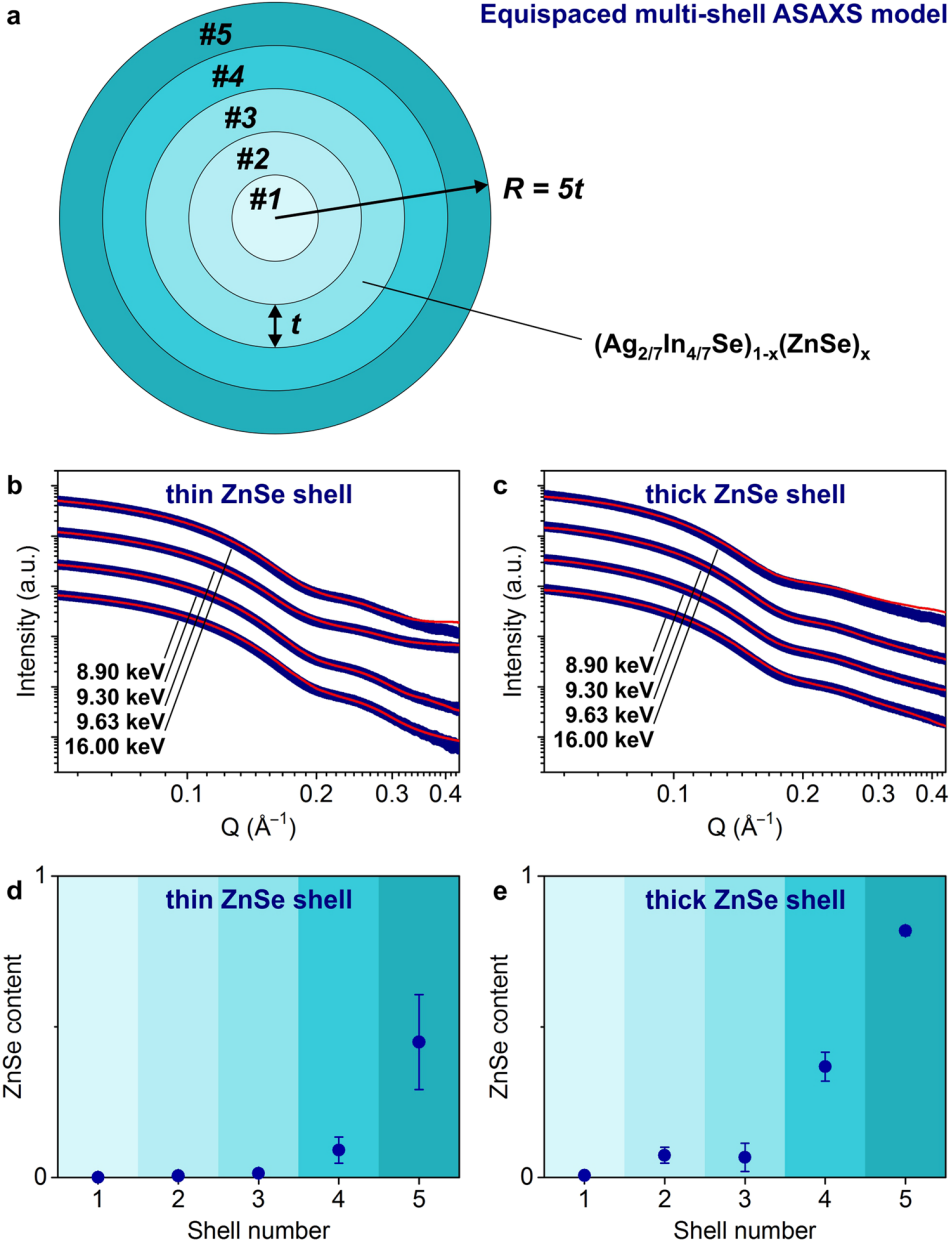
Anomalous SAXS measurements and fitting model. (**a**) Schematics of the equispaced 5-shell ASAXS model, (**b**,**c**) multi-energy ASAXS spectra, fitted with the model (offset for clarity), and (**d**,**e**) extracted radial distribution of ZnSe for Ag-In-Se/ZnSe core/shell nanocrystals. Data for thin ZnSe shell sample are shown in left panels and for thick ZnSe shell sample in right panels.

We choose to fit 5 shells, as illustrated in Fig. 3a. This provides sub-nanometer radial resolution of ZnSe gradients (*i.e*., 0.39 nm for thin ZnSe shell NCs and 0.41 nm for thick ZnSe shell NCs), thicknesses on the order of single atomic monolayer in this crystal structure^20^. Figure 3b,c show the measured scattering spectra and the fitting results for the thin-shell and thick-shell Ag-In-Se/ZnSe NCs. Despite the small deviations at larger Q-values, the fitting curves agree well with the measured data in the range of scattering vectors between 0.05 Å^–1^ and 0.45 Å^–1^ (*i.e*., in the oscillating part of spectra). Importantly, our simple equispaced 5-shell ASAXS model, for which the radial ZnSe distribution is the only fitting parameter, correctly matches the first minima of the scattering curves for both samples. This validates that, if carefully measured, the size, size distribution and composition can be determined from electron microscopy analysis and used as fixed input parameters in the ASAXS model.

Figures 3d,e plot extracted ZnSe radial distributions for graded Ag-In-Se/ZnSe core/shell NCs. For both samples, we observe a concentration gradient of the ZnSe shell. The ZnSe content decreases rapidly towards the center of NCs. Comparing two samples, the thin-shell NCs contain smaller amounts of ZnSe in each shell. The outermost layer of thin-shell Ag-In-Se/ZnSe NCs consists of equal amounts of Ag-In-Se and ZnSe, whereas the surface of thick-shell sample is nearly completely capped with ZnSe material. For thick-shell Ag-In-Se/ZnSe NCs, ZnSe exhibits higher degree of alloying, remaining only small central part as pure ternary Ag-In-Se composition. For the thin ZnSe shell NCs, however, three inner shells contain negligible amounts of ZnSe (Fig. 3d,e).

We explain the difference between two ZnSe profiles by the temperature dependence of solid-state diffusion of Zn. The cation-exchange mechanism of the Ag-In-Se/ZnSe core/shell growth occurs *via* diffusion of Zn atoms into the Ag-In-Se material and replacement of Ag and In cations^16^. Since diffusion processes are accelerated at higher temperatures, it is expected that the thick-shell Ag-In-Se/ZnSe NCs prepared at 150 °C, will show deeper penetration of ZnSe than thin-shell Ag-In-Se/ZnSe NCs, prepared at 50 °C. We analyze this Zn diffusion in more details in the Discussion section.

### Shape of Graded Ag-In-Se/ZnSe Core/Shell Nanocrystals

Above, the NCs were assumed to be spherical^16^. However, careful inspection of TEM images reveals an elongated shape for many NCs in the batch (Fig. 2d–f and h). For instance, for thick-shell Ag-In-Se/ZnSe NCs, the shape of the NCs can be estimated to prolate ellipsoid with major and minor radii of *R*_*major*_ = 4.3 nm, *R*_*minor*_ = 3.6 nm (Figure S4). Accordingly, we modify the fitting model and assume the prolate ellipsoidal shape of graded Ag-In-Se/ZnSe NCs. We denote the length of two minor axes as R and the major axis as *bR* (where *b* is a ratio between *R*_*major*_ and *R*_*minor*_) and take other input parameters as those of the spherical 5-shell ASAXS model. Comparison of the fitting results (Figure S5) reveals that the assumption of a spherical scatterer fits better in the oscillating part *(i.e*., at the first minimum), which is related to the location of the Zn, whereas the assumption of an ellipsoidal morphology of NCs shows a better agreement at small Q-values. From this, we conclude that the shape of Ag-In-Se/ZnSe core/shell NCs is indeed slightly elongated; however, the ZnSe distribution can be well described by a centrosymmetric radial gradient.

To provide detailed description of the shape of graded Ag-In-Se/ZnSe NCs, we employ an *ab initio* shape-retrieval method^15,21^. This approach analyzes SAXS spectra making no *a priori* assumptions related to the scatterer shape^22^. The average shape of nanocrystal scatterer is instead formed by “artificial dummy atoms” (called beads), which have defined diameter. The beads are allowed to move freely in order to fit the experimental SAXS patterns. The fitting procedure is repeated several times and all shapes are averaged. Finally, the probability of finding a single bead at the exact same positions (*i.e*., the occupancy) is obtained as a 3-dimensional map. Such occupancy numbers depend on the internal electron density of the particle as well as on the size distribution, thus representing the shape of NCs^15^.

**Figure 4:**
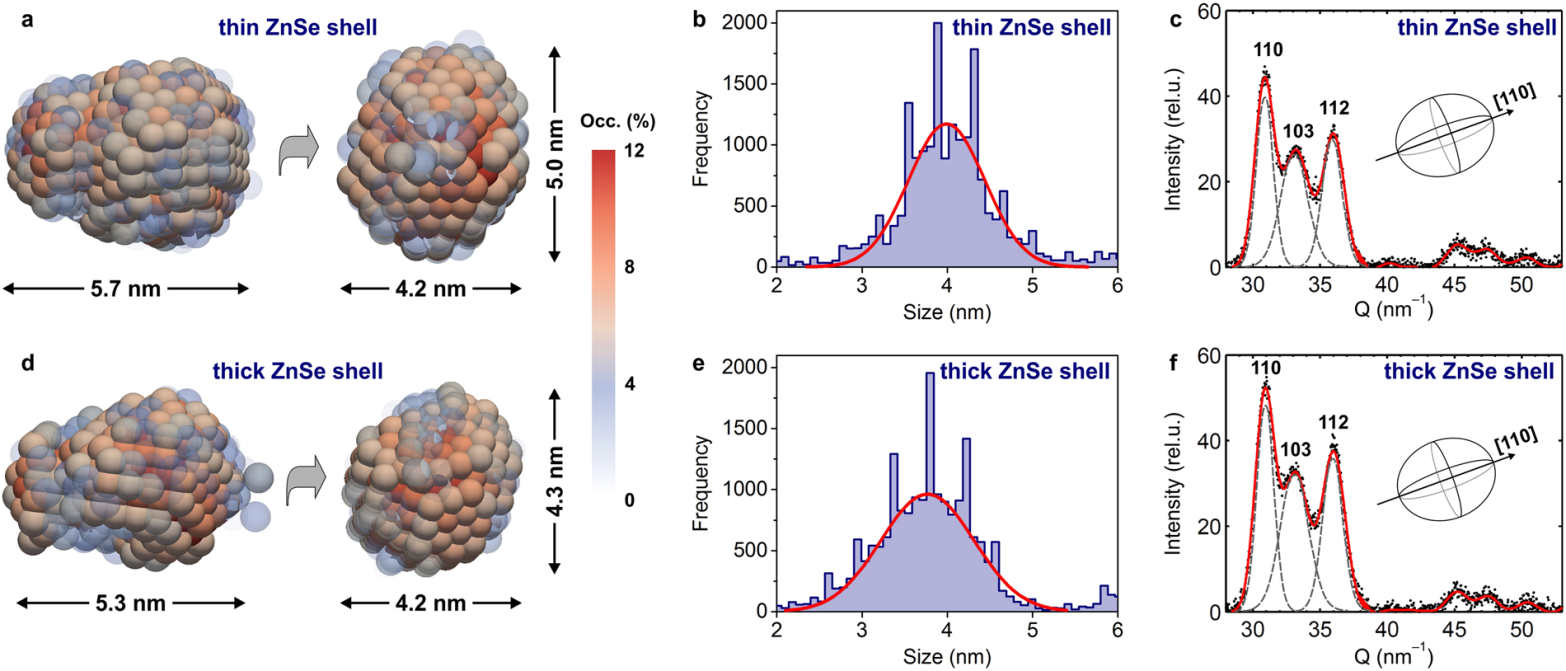
Shape and crystal structure of nanocrystal scatterer. (**a**,**d**) Average shapes and (**b**,**e**) diameter distributions of Ag-In-Se/ZnSe core/shell nanocrystals, as derived from the ab initio shape-retrieval SAXS method. (**c**,**f**) Wide angle X-ray scattering spectra of Ag-In-Se/ZnSe core/shell nanocrystals, shown together with Gaussian fits of their crystalline Bragg reflections. Data for thin ZnSe shell sample are shown in upper panels and for thick ZnSe shell sample in lower panels.

The average shapes of the Ag-In-Se/ZnSe NCs are derived from the occupancy maps in Fig. 4a,d. The elongated morphology of these average NC shapse comes from the overlaid distribution of more spherical and more ellipsoidal NCs that exist in the same sample (see TEM images in Figure S4). The average thick-shell Ag-In-Se/ZnSe NCs can be described as prolate ellipsoid with longer axis exceeding 5 nm and a ratio between major and minor radii ΔR = R_major_/R_minor_ = 1.3, whereas the thin shell NCs exhibit slightly larger aspect ratio ΔR = 1.4. Due to the finite size-distributions of the nanocrystal ensemble (10–12%, Fig. 2g and i) the dimensions of the NCs, shown in Fig. 4a and d, are known to be slightly overestimated, depending on the cut-off occupancy value^15^.

An unbiased size distribution analysis can be achieved using 10^4^ arbitrary line cuts through the average shapes of NCs (Fig. 4b and e). The diameters of the NCs are derived from the distances between the half-height occupancy values (Figure S6). Obtained size histograms show a good agreement with the electron microscopy measurements (Fig. 2g and i) and are centered around 3.8–4.0 nm for the thin-shell and thick-shell Ag-In-Se/ZnSe NCs. From the line cuts (Figure S6), we can calculate the maximum aspect ratio values, which confirm that the thin-shell NCs are slightly more elliptical (ΔR = 1.5) than the thick-shell Ag-In-Se/ZnSe NCs (ΔR = 1.2). The comparison between size distribution statistical analysis with the visual inspection of the retrieved mean 3D shapes confirms that bead models, shown in Fig. 4a and d, describe the characteristic ellipsoidal shape, although overestimating the absolute diameter values.

### Crystal Structure of Graded Ag-In-Se/ZnSe Core/Shell Nanocrystals

To determine the crystal structure of Ag-In-Se/ZnSe core/shell NCs, we analyze the wide-angle X-ray scattering (WAXS) spectra (Figure S7). The crystal structure of Ag-In-Se/ZnSe core/shell NCs belongs to the hexagonal wurtzite-type structure. The core-only Ag-In-Se NCs exhibit same crystal structure^16^, which confirms that the cation-exchange shell growth process does not substantially affect the anionic sublattice^7,23,24^.

Due to the small size of the Ag-In-Se/ZnSe core/shell NCs, Bragg reflections broaden and overlap. Dimension-specific peak broadening can be determined for the 110, 103, and 112 crystallographic directions (Fig. 4c and f). The crystal size in specific directions is calculated *via* Scherrer formula, assuming Gaussian broadening of Bragg reflections^25^. For the thin-shell Ag-In-Se/ZnSe NCs, calculated size values are D_110_ = 3.8 nm, D_103_ = 2.7 nm, and D_112_ = 3.3 nm, and, for the thick-shell Ag-In-Se/ZnSe NCs, D_110_ = 3.7 nm, D_103_ = 2.8 nm, and D_112_ = 3.1 nm (Fig. 4c and f). These crystallite size values are close to those extracted from the *ab initio* SAXS shape-retrieval and TEM analysis (Fig. 2g,i, 4b and e), suggesting that a major axis of ellipsoidal Ag-In-Se/ZnSe core/shell NCs is likely along the [110] crystallographic direction. Furthermore, we obtain good quantitative agreement between WAXS and SAXS analysis, specifically (i) similar overall crystallite size for thin-shell and thick-shell Ag-In-Se/ZnSe NCs and (ii) lower aspect ratio value for the thick ZnSe shell sample, indicating its slightly more spherical shape.

### Atomic Reconstructions of Graded Ag-In-Se/ZnSe Core/Shell Nanocrystals

**Figure 5:**
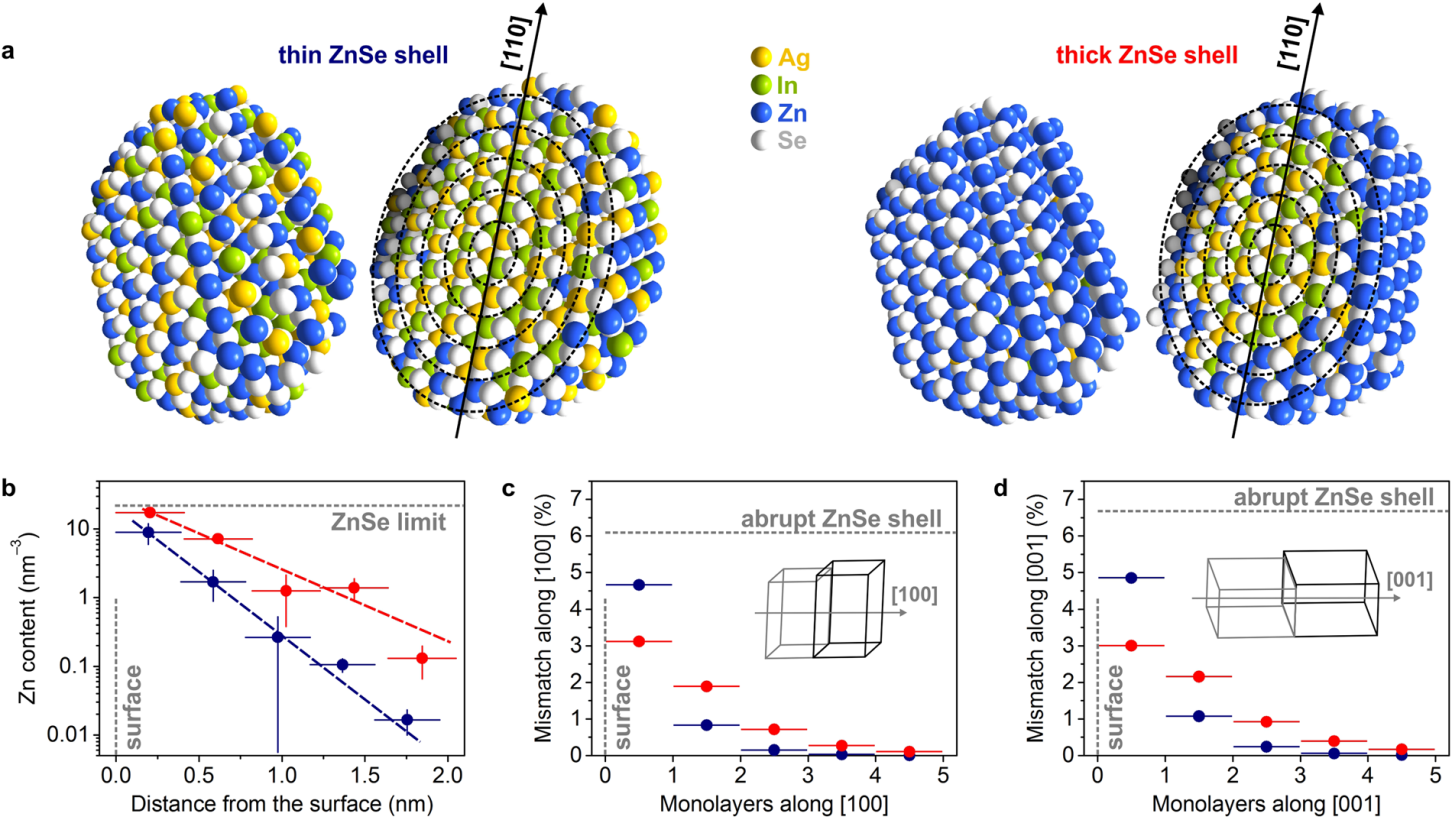
Structural analysis of graded Ag-In-Se/ZnSe core/shell nanocrystals. (**a**) Atomic reconstructions of Ag-In-Se/ZnSe core/shell nanocrystals, combining small- and wide-angle X-ray scattering results. The 5-shell ASAXS model is shown schematically on top of the reconstructions. (**b**) Radial Zn depth profile and (**c**,**d**) lattice mismatch calculations in [100] and [001] directions for the two Ag-In-Se/ZnSe core/shell nanocrystals. Data for thin ZnSe shell sample are shown in dark blue and for thick ZnSe shell sample in red. The lattice mismatch of an abrupt Ag-In-Se and ZnSe interface is illustrated in (**c**,**d**).

Combining the findings from ASAXS, SAXS, and WAXS, we obtain atomic reconstructions of thin- and thick-shell Ag-In-Se/ZnSe core/shell NCs (Fig. 5a). NCs have wurtzite-type crystal structure, outer dimensions as derived from the *ab initio* shape-retrieval SAXS analysis, and their largest axis along [110] direction. Reconstructed NCs are further split into 5 shells and for each shell, Ag and In atoms are substituted by Zn atoms to approach calculated ZnSe contents, so that the compositional gradient is consistent with our findings from the *equispaced multi-shell ASAXS model*.

Such accurate atomic reconstructions of NCs provide not only convenient visualization, but are necessary for theoretical studies using tight binding simulations, density functional theory, *ab initio* molecular dynamics^26–28^. In the discussion below, we show that the atomic reconstructions enable direct comparison between the two different shell syntheses and give insight into the relative performance of the resulting thin-shell and thick-shell Ag-In-Se/ZnSe core/shell NCs.

## Discussion

Detailed structural description of graded Ag-In-Se/ZnSe core/shell NCs enables quantitative studies of the solid-state Zn diffusion and the lattice strain at the core/shell interface. These results provide an explanation for the superior luminescence efficiencies of Ag-In-Se/ZnSe core/shell NCs capped with a thick ZnSe shell. Our data suggest that the optimal design for the graded core/shell NCs comprises full shell coverage of the outermost monolayer, which leads to minimal lattice strain and provides good surface protection.

### Solid-State Zn Diffusion in Ag-In-Se Nanocrystals

Solid-state diffusion processes are well-studied for many interfaces and thin films^29^. For NCs, these solid-state diffusion concepts are taken to qualitatively explain for example the Kirkendall effect or alloying^24,30,31^. Similarly, the diffusion processes, which govern the cation-exchange synthesis of Ag-In-Se/ZnSe core/shell NCs, are not yet quantified.

To study the solid-state diffusion of Zn in the Ag-In-Se NCs, the radial ZnSe profiles (Fig. 3d,e) are converted to the atomic density of Zn *vs*. distance from the surface (Fig. 5b, calculation details in the Supplementary Information). The Zn radial distributions show exponential profiles for both thin- and thick-shell Ag-In-Se/ZnSe NCs and can be fitted with a simple exponential decay function *F(x) = exp(C*_1_ *+ C*_2_ *x)*. Toward the surface of the NCs, the Zn atomic densities approach the ZnSe limit (≈22 Zn nm^–3^). However, the Zn content for thin-shell Ag-In-Se/ZnSe NCs is systematically lower than for the thick ZnSe shell sample. The outermost monolayer for the thin ZnSe shell NCs still contains sufficient amounts of Ag and In atoms, while the thick ZnSe shell provides full Zn coverage of the nanocrystal surface. Finally, the concentration gradient of Zn in the thin-shell Ag-In-Se/ZnSe NCs is more abrupt, indicated by the steeper nature of the fitting function.

We apply Fick’s first law to quantify the solid-state Zn diffusion in the Ag-In-Se NCs. We denote the Zn diffusivities as ${D}_{Zn}^{50}$ ^°C^ and ${D}_{Zn}^{150}$ ^°C^, reflecting the shell growth temperatures for thin-shell and thick-shell Ag-In-Se/ZnSe core/shell NCs. Calculated Zn diffusivity at 150 °C is >30% higher, which confirms more efficient cation-exchange process for the thick-shell Ag-In-Se/ZnSe NCs (${D}_{Zn}^{150}$ ^°C^ = 1.57·10^−17^ cm^2^·s^−1^ and ${D}_{Zn}^{50}$ ^°C^ = 1.18·10^−17^ cm^2^·s^−1^). The temperature dependence of Zn diffusion constants provides an estimate for the activation energy of the solid-state Zn diffusion in Ag-In-Se NCs (see details on calculation in the Supplementary Information). Comparing the Zn diffusivity values with the literature data^32^, the Zn diffusion in the Ag-In-Se NCs exhibits similar values to those for Zn diffusion through polycrystalline Cu-In-Ga-Se thin films at 150–200 °C (Figure S8). However, the activation energy of the Zn diffusion in Ag-In-Se NCs is notably smaller (*E*_*a*_ = 3.3 kJ·mol^−1^ for NCs and *E*_*a*_ = 119.6 kJ·mol^−1^ for thin films)^32^. This observation suggests that the first few atomic monolayers are easily permeable for the Zn atoms (*i.e*., the case of Ag-In-Se NCs), while micrometer-scale Zn diffusion costs significantly more energy (*i.e*., the case of Cu-In-Ga-Se thin films). Closer comparison for the graded Ag-In-Se/ZnSe NCs can be made with ZnTe/CdSe core/shell nanorods^33^. The energy barrier for the Cd diffusion in the isovalent ZnTe/CdSe NCs is estimated to 20 kJ·mol^−1^, 6 times higher than for the Ag-In-Se/ZnSe core/shell NCs. The presence of many cationic vacancies in the structure of I-III-VI materials causes accelerated diffusion^18^, eventually lowering the activation energy of the process. Small activation energy of the solid-state Zn diffusion in the Ag-In-Se NCs suggests that atomic rearrangement may occur even at close to ambient temperature conditions.

### Lattice Mismatch in Graded Ag-In-Se/ZnSe Core/Shell Nanocrystals

Certain amount of lattice mismatch appears at the interface between the core and the shell materials in the core/shell NCs^14^. For the case of wurtzite-type Ag-In-Se and ZnSe materials, a lattice mismatch of 6.1% along [100] and 6.7% along [001] directions is expected (Fig. 5c,d)^16,20^. A gradient distribution of ZnSe leads to smooth change of unit cell parameters over several monolayers, which minimizes the lattice stresses. For graded Ag-In-Se/ZnSe core/shell NCs, the largest lattice strain appears between two outermost monolayers. It is, however, significantly lower, comparing to the abrupt core/shell morphology. The largest lattice misfit for the thin-shell Ag-In-Se/ZnSe NCs is below 5%, while the mismatch profile is even lower for the thick-shell Ag-In-Se/ZnSe core/shell NCs. Owing to full ZnSe coverage of the outermost layer and relatively large content of ZnSe in the layer directly beneath, the lattice strain for the thick ZnSe shell NCs is approximately 3% in both [100] and [001] directions (calculation details in the Supplementary Information). The 3%-mismatch allows effective lattice relaxation without formation of large number of interface defects^23^. The elimination of interfacial defects may further explain the superior luminescent properties of graded Ag-In-Se/ZnSe core/shell NCs, capped with thick ZnSe shell.

In conclusion, the approach presented here enables the quantitative reconstruction of the atomic structure of functional nanomaterials with complex composition or doping profiles. Such reconstructions can drive nanoscience forward: they enable us to understand the reaction mechanisms and diffusion occurring in different synthesis approaches, they are needed for first principles computational investigations, which are now becoming possible on realistically-sized NCs consisting of hundreds of atoms, and they can be used to obtain detailed understanding of the optical, electronic, and vibrational properties of the NCs. Exploring these structure-property relationships paves the way for rational design of nanomaterials and facilitate improved performance of NC-based luminescent materials *via* sophisticated engineering of graded core/shell interface.

## Methods

### Synthesis of materials

Ag-In-Se NCs with Ag:In atomic ratio of ~0.6 were prepared *via* an amide-promoted synthesis, according to ref.^16^. Their exact composition was measured by EDX spectroscopy (19.8 at.% Ag; 31.5 at.% In; 48.7 at.% Se). These Ag-In-Se NCs were used for both Ag-In-Se/ZnSe core/shell NC batches.

The growth of ZnSe shell was carried out according to ref.^34^. Briefly, for the synthesis of graded Ag-In-Se/ZnSe core/shell NCs, 17 mg of core-only Ag-In-Se NCs were re-dissolved in 12 mL of 0.17 M TOP:Se (*i.e*., Se solution in tri-*n*-octylphosphine, 2 mmol of Se in total). The mixture was rapidly heated to the reaction temperature of 50 °C to grow the thin ZnSe shell or to 150 °C for thick ZnSe shell formation. A set reaction temperature was then maintained while 6 mL of TOP solution containing 0.5 mmol of diethylzinc and 0.5 mmol of Se (in form of TOP:Se) was added with the rate of 0.6 mL·min^–1^. Obtained Ag-In-Se/ZnSe core/shell NCs were purified 2 times, first by addition of toluene and second by addition methanol, followed each time by centrifugation and re-dispersion in toluene.

### Characterization and data analysis

Energy dispersive X-ray (EDX) spectroscopy was performed on a FEI Quanta 200 SEM microscope. TEM and high-resolution TEM images were taken on a Tecnai F30 TEM microscope. Size distribution TEM analysis was performed by measuring >100 NCs, using ImageJ software. For the ellipsoidal shape of NCs, two diameters were measured, including the longest dimension of NCs.

Small-angle and wide-angle X-ray scattering experiments (SAXS and WAXS) were performed at the MS beamline of the Swiss Light Source. Diluted colloidal solutions of Ag-In-Se/ZnSe core/shell NCs in toluene were loaded into glass capillaries and capped with epoxy resin. For anomalous small-angle X-ray scattering (ASAXS), four different energies of the incident X-ray beam were chosen: three (9.63 keV, 9.30 keV and 8.90 keV) in the vicinity of the K-absorption edge of Zn (9.659 keV) and one at a much higher energy (16.00 keV). Energies of the incident X-ray beam were chosen based on different absorption coefficients of Zn and the characteristics of the beamline. Measured ASAXS spectra were subtracted with reference background signals, originating from air, the capillary, solvent, and TOP ligand. The equispaced multi-shell ASAXS model was developed, using the *cftool* toolbox of MATLAB. To ensure the robustness of ASAXS results, the fitting procedure was repeated up to 15 times with scattering data arbitrarily selected from within the experimental error band each time. The error bars for the stoichiometry of each shell layer were obtained accordingly. The *ab initio* shape-retrieval SAXS analysis of NCs uses dummy atom models DAMMIN and DAMAVER, which are based on scattering calculations for the assembly of spheres with equal diameter^15^. The diameter of the displayed dummy atoms is 0.7 nm, and the accuracy of shape dimensions is ±0.4 nm. The shape-retrieval SAXS software is written in MATLAB. Atomic reconstructions of graded Ag-In-Se/ZnSe core/shell NCs were performed using Diamond structure visualization software.

## Electronic supplementary material

Supplementary Information
