# Supplementary material for: Mapping the Atomistic Structure of Graded Core/Shell Colloidal Nanocrystals
Source: Sci Rep. 2017 Sep 15;7:11718. doi: 10.1038/s41598-017-11996-2 (PMC5601428; doi:10.1038/s41598-017-11996-2)
Supplement: Supplementary file 1 — Supplementary Information [file 41598_2017_11996_MOESM1_ESM.pdf]

Supplementary Information for

Mapping the Atomistic Structure of Graded Core/Shell Colloidal Nanocrystals

Maksym Yarema,[1] Yunhua Xing,[1] Rainer T. Lechner,[2] Lukas Ludescher,[2] Nikola Dordevic,[1] Weyde M. M. Lin,[1] Olesya Yarema,[1] and Vanessa Wood[1,*]

[1] Laboratory for Nanoelectronics, Department of Information Technology and Electrical Engineering, ETH Zurich, CH-8092 Zurich, Switzerland.
[2] Institute of Physics, Montanuniversitaet Leoben, A-8700 Leoben, Austria
* Correspondence to vwood@ethz.ch

Calculations

Equispaced multi-shell ASAXS model

According to SAXS theory, the scattering intensity from a diluted suspension of particles can be expressed as

$$I(q) = \langle |F(q)|^2 \rangle \quad (1)$$

where *F(q)* and *q* are the form factor and the scattering vector. The form factor *F(q)* of a nanoparticle is a Fourier transform from real space to reciprocal space of the scattering length density[1]

$$F(q) = \int r_e \Delta\rho(R) e^{-iqR} dR \quad (2)$$

where $\Delta\rho(R)$ is the electron density contrast between the particle and the matrix materials at position *R* and r_e is the classical electron radius. For a homogeneous particle with spherical symmetry, the scattering intensity can be analytically written as

$$I(q) = \left(4\pi R^3 r_e \Delta\rho \frac{sin(qR) - qRcos(qR)}{(qR)^3}\right)^2 \quad (3)$$

with R as the radius of spherical scatterer. For more geometrically sophisticated particles, direction components of *q* need to be averaged, and thus the scattering intensity might only be able to be numerically calculated.
In the proximity of their absorption edge energies, atomic scattering factors receive energy-dependent terms. The atomic scattering factor can be expressed as

$$f(Z,E) = f_0(Z) + f'(E) + if''(E) \quad (4)$$

where $f_0(Z)$ is simply the atomic number of the anomalous scatterer and the others are resonance-induced terms, which are related to each other by the Kramer-Kronig relation (Figure S1).[2] Consequently, additional energy-dependent electron density are introduced to the conventional electron density of the nanocrystal by the anomalous scatterer and the scattering intensity, *I(q,E)*, needs to be modified as a quadratic equation, containing both, non-resonant and resonant form factors, $F_0(q)$ and $F_R(q)$:

$$I(q,E) = F_0^2(q) + 2f'(E)F_0(q)F_R(q) + |f'(E)^2 + f''(E)^2|F_R^2(q). \quad (5)$$

A method, which uses energy-dependent scattering to reveal the atomic structure of the material, is often referred as anomalous SAXS (i.e., ASAXS).

Finally, finite size distribution, *G(R)*, of nanocrystal scatterer should be taken into account, defining the ASAXS scattering intensity as

$$I(q,E) = \int_0^\infty G(R)[F_0^2(q) + 2f'(E)F_0(q)F_R(q) + |f'(E)^2 + f''(E)^2|F_R^2(q)]dR. \quad (6)$$

For the multi-shell electron density model, we use the linearity property of the Fourier transform.[1] To eliminate geometrical fitting parameters, we fix an equal thickness for each shell. Under these conditions, the non-resonant form factor can be expressed as

$$F_0(q) = 4\pi r_e \left[R^3(\rho_k - \rho_{solv})\frac{sin(qR) - qRcos(qR)}{(qR)^3} + (R-t)^3(\rho_{k-1} - \rho_k)\frac{sin(q(R-t)) - q(R-t)cos(q(R-t))}{(q(R-t))^3} + \cdots\right] \quad (7)$$

where *k* is the number of shells, ρ_k and ρ_{solv} is the total electron density in each shell and in solvent (toluene), *t* is the thickness of the shell, and the radius of spherical scatterer can be calculated as $R = k \cdot t$. The resonant form factor can be expressed in the same way by using the atomic densities of anomalous scatterer (Zn atom in our case), $N_{A,k}$, instead of ρ_k in the (7):

$$F_R(q) = 4\pi r_e \left[R^3 N_{A,k}\frac{sin(qR) - qRcos(qR)}{(qR)^3} + (R-t)^3(N_{A,k-1} - N_{A,k})\frac{sin(q(R-t)) - q(R-t)cos(q(R-t))}{(q(R-t))^3} + \cdots\right]. \quad (8)$$

ZnSe profiles in the Ag-In-Se/ZnSe core/shell nanocrystals

Assuming the total composition of each shell as a solid solution between In-rich Ag-In-Se (Ag:In ratio is 2) and ZnSe, we can describe it with the formula $(Ag_{2/7}In_{4/7}Se)_{1-x}(ZnSe)_x$ having variable content of ZnSe, *x*, in each shell.
The total electron density, ρ, can be calculated as a ratio between the number of electrons and the volume of the unit cell ($N_{e,unit}$ and V_{unit}, respectively):

$$\rho = \frac{N_{e,unit}}{V_{unit}}. \quad (9)$$

The volume of the unit cell with the composition $(Ag_{2/7}In_{4/7}Se)_{1-x}(ZnSe)_x$ can be derived from

$$V_{unit} = \frac{un_{unit} M_{w,(Ag_{2/7}In_{4/7}Se)_{1-x}(ZnSe)_x}}{d_{(Ag_{2/7}In_{4/7}Se)_{1-x}(ZnSe)_x}} \quad (10)$$

where $d_{(Ag_{2/7}In_{4/7}Se)_{1-x}(ZnSe)_x}$ and $M_{w,(Ag_{2/7}In_{4/7}Se)_{1-x}(ZnSe)_x}$ are mass density and molecular weight of

$(Ag_{2/7}In_{4/7}Se)_{1-x}(ZnSe)_x$, u is unified atomic mass unit, $1.66054 \cdot 10^{-24}$ g, and n_{unit} is the number of molecular units in the unit cell (here $n_{unit} = 2$). Substituting (10) in (9) relates total electron density to the ZnSe content, x, in the composition as

$$\rho = \frac{N_{e,(Ag_{2/7}In_{4/7}Se)_{1-x}(ZnSe)_x} d_{(Ag_{2/7}In_{4/7}Se)_{1-x}(ZnSe)_x}}{uM_{w,(Ag_{2/7}In_{4/7}Se)_{1-x}(ZnSe)_x}} \quad (11)$$

where $N_{e,(Ag_{2/7}In_{4/7}Se)_{1-x}(ZnSe)_x}$ is the number of electrons in the $(Ag_{2/7}In_{4/7}Se)_{1-x}(ZnSe)_x$ unit. Equation (11) is plotted in Figure S2.

Since the anionic sublattice (i.e., Se skeleton) is not substantially affected by the cation-exchange shell growth process (confirmed by the WAXS spectra), it is rational to assume mass density of $(Ag_{2/7}In_{4/7}Se)_{1-x}(ZnSe)_x$ as the linear interpolation between densities of In-rich Ag-In-Se and ZnSe materials (5.385 g·cm^{-3} and 5.26 g·cm^{-3}, respectively).[3, 4] All terms in (11) can be expressed as functions of ZnSe content, x:

$$d_{(Ag_{2/7}In_{4/7}Se)_{1-x}(ZnSe)_x} = 5.385(1-x) + 5.27x \quad (12)$$

$$N_{e,(Ag_{2/7}In_{4/7}Se)_{1-x}(ZnSe)_x} = (1-x)\left(\frac{2}{7}Z_{Ag} + \frac{4}{7}Z_{In}\right) + xZ_{Zn} + Z_{Se} \quad (13)$$

$$M_{w,(Ag_{\frac{2}{7}}In_{\frac{4}{7}}Se)_{1-x}(ZnSe)_x} = (1-x)\left(\frac{2}{7}M_{w,Ag} + \frac{4}{7}M_{w,In}\right) + xM_{w,Zn} + M_{w,Se} \quad (14)$$

where $Z_{Ag} = 47$, $Z_{In} = 49$, $Z_{Zn} = 30$, $Z_{Se} = 34$, $M_{w,Ag} = 107.87$, $M_{w,In} = 114.82$, $M_{w,Zn} = 65.38$, and $M_{w,Se} = 78.96$.

Similarly, the Zn-specific atomic density, N_{Zn}, can be as a ratio between number of zinc atoms in the unit cell and its volume. Using (10), the Zn atomic density can be related to the ZnSe content, x, in the composition $(Ag_{2/7}In_{4/7}Se)_{1-x}(ZnSe)_x$:

$$N_{Zn} = \frac{x d_{(Ag_{2/7}In_{4/7}Se)_{1-x}(ZnSe)_x}}{uM_{w,(Ag_{2/7}In_{4/7}Se)_{1-x}(ZnSe)_x}}. \quad (15)$$

Equation (15) is plotted in Figure S3.

Using relations (11-15) allows us to relate both non-resonant and resonant form factors to the ZnSe content x, thus minimizing the number of fitting parameters for (6-8) to k (i.e., number of fitting shells, $k = 5$ in this study). We also introduce a global scaling factor C to the fitting equation (6) as another fitting parameter, in order to account for systematic errors, including errors due to collimation and calibration.

Solid-state Zn diffusion in the Ag-In-Se nanocrystals

Zinc diffusivities are calculated from the depth profiles. From the multi-shell ASAXS model with 5 shells and with the radii of nanocrystals from distribution functions (Figures 2g and 2i; $R_{thin\text{-}shell} = 1.95$ nm, $R_{thick\text{-}shell} = 2.05$ nm), shell thicknesses can be calculated as 0.39 nm for thin-shell and 0.41 nm for thick-shell Ag-In-Se/ZnSe core/shell nanocrystals. The ZnSe contents, x, of each shell are associated with the value at the middle of each shell (i.e., at following distances from the surface, r: 0.1R, 0.3R, 0.5R, 0.7R, and 0.9R). The fitting function of the Zn atomic density, N_{Zn}, and the distance from the surface, r, is

$$N_{Zn} = e^{(C_1 + C_2 r)} \quad (16)$$

where $C_1 = 3.02$ and $C_2 = -4.28$ for thin ZnSe shell sample and $C_1 = 3.36$ and $C_2 = -2.41$ for thick ZnSe shell sample. These values can be used to determine the diffusivity of Zn atoms for a given synthesis temperature.

To do so, we assume the diffusivity of Zn atoms, D_{Zn}, can be calculated from the Fick's first law of diffusion:[5]

$$J_{Zn} = -D_{Zn}\frac{dN_{Zn}}{dr} \quad (17)$$

where J_{Zn} is the diffusion flux. The term $\frac{dN_{Zn}}{dr}$ can be calculate from (16) and at the surface of the nanocrystal ($r = 0$), it can be simplified to

$$\frac{dN_{Zn}}{dr} = C_2 e^{C_1} e^{C_2 r}|_{r=0} = C_2 e^{C_1}. \quad (18)$$

The rate at which the Zn concentration decreases from the nanocrystal surface towards the core is therefore 88 atoms·nm^{-1} for thin ZnSe shell sample and 69 atoms·nm^{-1} for thick ZnSe shell sample.

For reaction-limited conditions, diffusion flux, J_{Zn}, is the total amount of Zn precursor, $[ZnEt_2]_0$, exposed to the total surface area of nanocrystals, A_{tot}, over reaction time, t_{reac}:

$$J_{Zn} = \frac{[ZnEt_2]_{tot}}{A_{tot} t_{reac}}. \quad (19)$$

The total surface area of nanocrystals can be calculated as

$$A_{tot} = A_{NC}\frac{m_{tot}}{m_{NC}} \quad (20)$$

where A_{NC} and m_{NC} are the surface area and the mass of single nanocrystal, m_{tot} is the total mass of introduced nanocrystals. Assuming spherical shape of Ag-In-Se nanocrystals, the diffusion flux equation (20) can be modified to

$$J_{Zn} = \frac{[ZnEt_2]_{tot} d_{Ag_{2/7}In_{4/7}Se} R}{3 m_{tot} t_{reac}}. \quad (21)$$

where all initial parameters are known: $[ZnEt_2]_{tot} = 3.01 \cdot 10^{20}$ (i.e., 0.5 mmol), $m_{tot} = 0.017$ g, $d_{Ag_{2/7}In_{4/7}Se} = 5.385$ g·cm^{-3}, and $t_{reac} = 600$ s.

Because thin-shell and thick-shell Ag-In-Se/ZnSe nanocrystals are prepared at different temperatures (50°C and 150°C, respectively), we denote diffusion fluxes as $J_{Zn}^{50°C}$ and $J_{Zn}^{150°C}$. From (21), diffusion fluxes are equal to $J_{Zn}^{50°C} = 0.1033$ nm^{-2}s^{-1} and $J_{Zn}^{150°C} = 0.1086$ nm^{-2}s^{-1}.

We thus determine Zn diffusivities for two reaction temperatures: $D_{Zn}^{50°C} = 1.18 \cdot 10^{-17}$ cm^{2}s^{-1} and $D_{Zn}^{150°C} = 1.57 \cdot 10^{-17}$ cm^{2}s^{-1}

Assuming Arrhenius-type temperature dependence of Zn diffusivity,[6]

$$D_{Zn} = D_0 e^{\left(-\frac{E_{a,Zn}}{k_B T}\right)} \quad (22)$$

activation energy, $E_{a,Zn}$, can be estimated from the ratio of Zn diffusivities at 150°C and 50°C. The activation energy for the Zn diffusion in the Ag-In-Se nanocrystals is equal to 3.3 kJ·mol^{-1} (or 34 meV).

Lattice mismatch in the Ag-In-Se/ZnSe core/shell nanocrystals

The unit cell parameters, a and c, of $(Ag_{2/7}In_{4/7}Se)_{1-x}(ZnSe)_x$ are calculated as a linear interpolation between lattice constants for the wurtzite-type modifications of In-rich Ag-In-Se (a = 0.4232 nm, c = 0.6972 nm)[3] and ZnSe (a = 0.3974 nm, c = 0.6506 nm):[7]

$$a_{(Ag_{2/7}In_{4/7}Se)_{1-x}(ZnSe)_x} = 0.4232(1-x) + 0.3974x \qquad (23)$$

$$c_{(Ag_{2/7}In_{4/7}Se)_{1-x}(ZnSe)_x} = 0.6972(1-x) + 0.6506x \qquad (24)$$

The lattice mismatch is calculated between each two atomic monolayers along two crystallographic directions, [100] and [001] (i.e., a and c directions, respectively). The monolayer thickness along [100] direction is estimated as the unit cell parameter a, while along the [001] direction as a half of the unit cell parameter c ($1ML_{[100]} \approx 0.4$ nm, $1ML_{[001]} \approx 0.35$ nm). The content of ZnSe, x, is calculated from (17) at following distances from the surface: at r = 0, 0.4, 0.8, 1.2, 1.6, and 2.0 for mismatch calculations along the [100] direction, and at r = 0, 0.35, 0.70, 1.05, 1.40, and 1.75 for mismatch calculations along [001] direction. The upper limit of $N_{Zn,max}$ = 21.98 nm^{-3} is applied for the surface monolayer of thick ZnSe shell sample (Figure S4). The unit cell parameters are defined from (24-25) and the lattice mismatches are calculated as

$$\Delta a_i = \frac{a_i - a_{i-1}}{a_i} \cdot 100\% \qquad (25)$$

$$\Delta c_i = \frac{c_i - c_{i-1}}{c_i} \cdot 100\% \qquad (26)$$

where Δa_i and Δc_i are the lattice mismatches along [100] and [001] directions, respectively, and i is the number of monolayer from the surface.
The lattice mismatches are also calculated between the $Ag_{2/7}In_{4/7}Se$ and ZnSe materials (i.e., upper mismatch limit for the abrupt-shell $Ag_{2/7}In_{4/7}Se$/ZnSe core/shell nanocrystals).

Supporting Figures

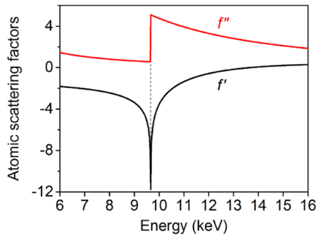

Figure S1. Energy-dependent correction terms of atomic scattering factor of Zn. Real part and imaginary part of the atomic scattering factor of Zn are depicted. Anomalous effects can be observed near the K-absorption edge (9.659 keV) highlighted by the dashed line.

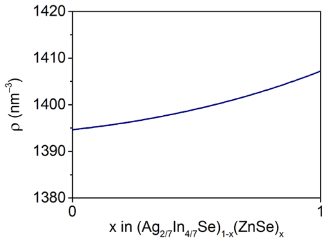

Figure S2. Total electron density as a function of ZnSe content, x, in $(Ag_{2/7}In_{4/7}Se)_{1-x}(ZnSe)_x$ composition. Calculated from Eq. 11.

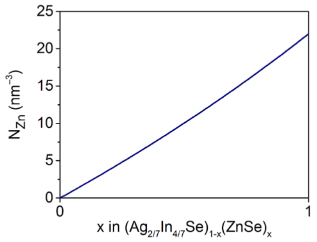

Figure S3. Zn atomic density as a function of ZnSe content, x, in $(Ag_{2/7}In_{4/7}Se)_{1-x}(ZnSe)_x$ composition. Calculated from Eq. 15.

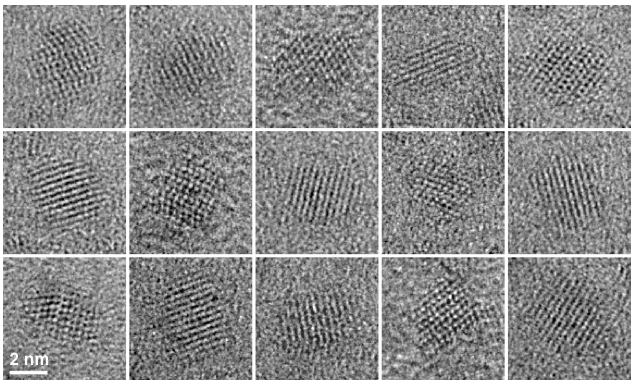

Figure S4. Selected high-resolution TEM images of thick-shell Ag-In-Se/ZnSe nanocrystals, revealing an ellipsoidal morphology with axes R_{major} = 4.2 ± 0.3 nm, R_{minor} = 3.4 ± 0.3 nm.

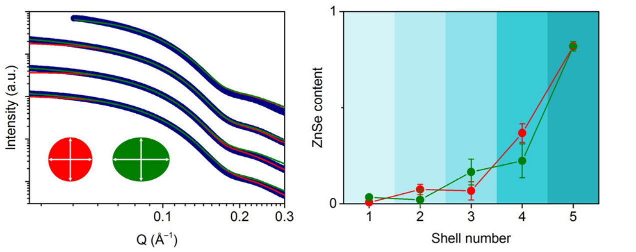

Figure S5. Comparison between multi-shell ASAXS model for spherical (fits and data in red) and ellipsoidal (fits and data in green) shape of scatterer.

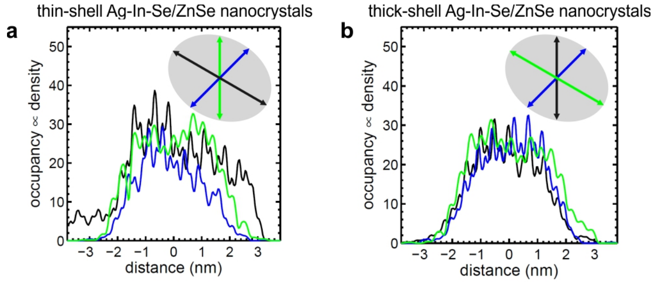

Figure S6 (A) Cuts through the centre of the 3D shapes shown in Figures 4a and 4d for the thin-shell and thick-shell Ag-In-Se/ZnSe NCs. The occupancy is plotted versus the distance from the centre of mass of the nanocrystal average shapes. The peaks in the cuts are due to single beads forming the shapes. The cuts are along three directions perpendicular to each other. The diameters $D_{1\text{-}3}$, and $D_{4\text{-}6}$ are derived from the distance between the half-height occupancy values.[8] The more inhomogeneous occupancy distribution of the thin ZnSe shell NCs suggests an inhomogeneous electron density along the different directions.

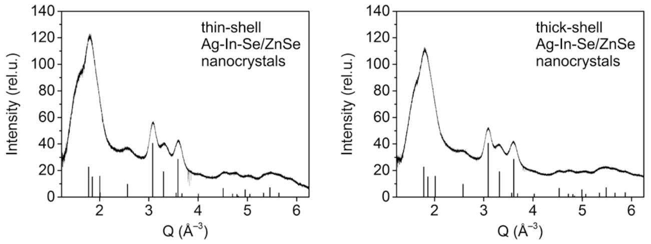

Figure S7. Wide-angle X-ray scattering of thin-shell and thick-shell Ag-In-Se/ZnSe nanocrystals with theoretical peak positions for wurtzite-type structure.

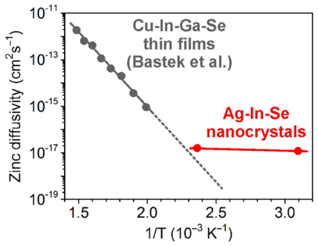

Figure S8. Diffusivities of Zn atoms through the Cu-In-Ga-Se thin films[6] and in Ag-In-Se nanocrystals.

References

1. Li, T.; Senesi, A. J.; Lee, B., Small Angle X-ray Scattering for Nanoparticle Research. *Chemical Reviews* **2016,** 116, 11128-11180.
2. Stuhrmann, H. B., Resonance scattering in macromolecular structure research. In *Characterization of Polymers in the Solid State II: Synchrotron Radiation, X-ray Scattering and Electron Microscopy*, Kausch, H. H.; Zachmann, H. G., Eds. Springer Berlin Heidelberg: Berlin, Heidelberg, **1985**; pp 123-163.
3. Yarema, O.; Yarema, M.; Bozyigit, D.; Lin, W. M. M.; Wood, V., Independent Composition and Size Control for Highly Luminescent Indium-Rich Silver Indium Selenide Nanocrystals. *ACS Nano* **2015,** 9, 11134-11142.
4. *Handbook on Physical Properties of Semiconductors: Volume 3: II–VI Compound Semiconductors*. Springer US: Boston, MA, **2004**; pp 161-210.
5. Cussler, E. L., *Diffusion: mass transfer in fluid systems*. Cambridge university press: **2009**.
6. Bastek, J.; Stolwijk, N. A.; Wuerz, R.; Eicke, A.; Albert, J.; Sadewasser, S., Zinc diffusion in polycrystalline Cu(In,Ga)Se_2 and single-crystal $CuInSe_2$ layers. *Applied Physics Letters* **2012,** 101, 074105.
7. Yeh, C.-Y.; Lu, Z. W.; Froyen, S.; Zunger, A., Zinc-blende-wurtzite polytypism in semiconductors. *Physical Review B* **1992,** 46, 10086-10097.
8. Burian, M.; Fritz-Popovski, G.; He, M.; Kovalenko, M. V.; Paris, O.; Lechner, R. T., Considerations on the model-free shape retrieval of inorganic nanocrystals from small-angle scattering data. *Journal of Applied Crystallography* **2015,** 48, 857-868.
